# External fixation reconstruction of the residual problems of benign bone tumours

**DOI:** 10.1007/s11751-016-0244-8

**Published:** 2016-02-12

**Authors:** Levent Eralp, F. Erkal Bilen, S. Robert Rozbruch, Mehmet Kocaoglu, Ahmed I. Hammoudi

**Affiliations:** Department of Orthopaedics and Traumatology, Istanbul Faculty of Medicine, Istanbul University, 34390 Topkapi, Istanbul, Turkey; Department of Orthopaedics and Traumatology, Memorial Health Group, 34385 Okmeydani, Istanbul, Turkey; Hospital for Special Surgery, Limb Lengthening and Complex Reconstruction Service (LLCRS), Weill Cornell Medical College, Cornell University, 535 East 70th Street, New York, NY 10021 USA; Orthopedic Department, Faculty of Medicine, Al-Azhar University Hospitals, Nasr City, Cairo, 11884 Egypt

**Keywords:** Benign bone tumours, External fixation, Limb reconstruction, Distraction osteogenesis, Shortening, Bone deformity

## Abstract

The mechanical features of and biologic response to using distraction osteogenesis with the circular external fixator are the unique aspects of Ilizarov’s contribution that allows deformity correction and reconstruction of bone defects. We present a retrospective study of 20 patients who suffered from a variety of benign tumours for which external fixators (EF) were used to treat deformity, bone loss, and limb-length discrepancy. A total of 26 bony segments in twenty patients (10 males, 10 females; mean age 17 years; range 7–58 years) were treated with EF for residual problems from the tumour itself (primary treatment) in 8 patients and for complications related to the primary surgery (secondary treatment) in 12 patients. Histological diagnoses were Ollier’s disease (*n* = 4), Fibrous Dysplasia (*n* = 5), Congenital multiple exostosis (*n* = 5), giant cell tumour (*n* = 2) and one case for chondromyxoid fibroma, desmoid fibroma, chondroma and unicameral bone cyst. Various types of external fixators used to treat these problems. These were Ilizarov, unilateral fixator, multiaxial correction frame (Biomet, Parsippany, NJ), Taylor spatial frame (Memphis, TN) and smart correction multiaxial frame. The mean follow-up time was 69.5 months (range 35–108 months). The mean external fixation time was 159.5 days (range 27–300 days). The mean external fixation index was 67.4 days/cm (12–610) in 26 limbs who underwent distraction osteogenesis. The mean length of distraction was 4.9 cm (range 0.2–14 cm). At final follow-up, all patients had returned to normal activities. Complications were in the form of knee arthrodesis in one patient, pin tract infection in six and residual shortening in eight patients. The use of EF and the principles of distraction osteogenesis, in the management of problems associated with benign bone tumours and related surgery yields successful results especially in young patients. With this approach, the risk for recurrence of shortening and deformity may be minimized with overcorrection or over-lengthening as dictated by preoperative planning.

## Introduction

The management of limb deformity, shortening and bone defects in the treatment of benign tumours is a major challenge [1, 2]. The radical and aggressive nature of surgical therapy has to be balanced with the treatment-related morbidity, i.e. complications, the need for reconstructive stabilization and potential functional deficit. The decision is a challenge for the orthopaedic surgeon [3]. Conventional methods of correcting deformity and limb-length inequality, such as shortening, single or multiple osteotomies or epiphysiodesis are limited in their scope and often unpredictable or unsatisfactory. Alternative methods of treating bone defects include free autograft, vascularized bone graft, allograft, artificial bone substitutes and prostheses [4]. However, these methods have disadvantages and a high incidence of complications. Long-term results can be unsatisfactory especially after resection of extensive or juxta-articular tumours [2, 5–7].

Ilizarov introduced the concept of induction of local bone formation with a minimally invasive procedure, the process he called distraction osteogenesis (DO) [8]. DO has been used widely to treat traumatic bone loss, nonunion, osteomyelitis, malunion, limb-length discrepancy and to correct deformity [9–12]. The method embraces biomechanical stability, minimally invasive surgery, regeneration of new bone with gradual lengthening of the soft tissues [5]. There are few studies of its use in the treatment of benign bone tumours [13, 14]. In this study we describe our experience of the use of external fixators to correct deformity, limb-length discrepancy, contractures and similar problems related to the primary treatment of benign bone tumours or for the secondary complications of other primary treatment.

## Materials and methods

Informed consent to participate in this study was obtained from all patients and the Institutional Review Board approved this study. External fixation techniques with or without intramedullary nailing (IMN) were used in 26 bony segments in 20 patients (10 males and 10 females) who had been treated for benign bone tumours and subsequently developed shortening, deformity or other complications. The reconstruction procedures were performed in two centres. The mean age at surgery was 17 years (7–58 years). Physical examination of the affected limb was complemented by plain radiography, computerized tomography and magnetic resonance imaging as necessary. The treatment was related to residual problems from the tumour itself (primary treatment) in 8 patients and for complications related to the primary surgery (secondary treatment) in 12 patients. All problems were either deformity or shortening, or both, or osteomyelitis.

Histological diagnoses included Ollier’s disease (OD) in 6 segments (four patients) (Figs. 1, 2, 3, 4, 5), fibrous dysplasia (FD) in 8 (five patients) (Figs. 6, 7, 8, 9, 10, 11, 12), congenital multiple exostosis (CME) in 6 (five patients) (Figs. 13, 14, 15, 16, 17, 18a, b), giant cell tumour (GCT) in 2 (two patients), desmoid fibroma (DF) in 1 (one patient), chondromyxoid fibroma (CMF) in 1 (one patient), chondroma in 1 (one patient) and unicameral bone cyst (UBC) in 1 (one patient) (Table 1).

**Fig. 1:**
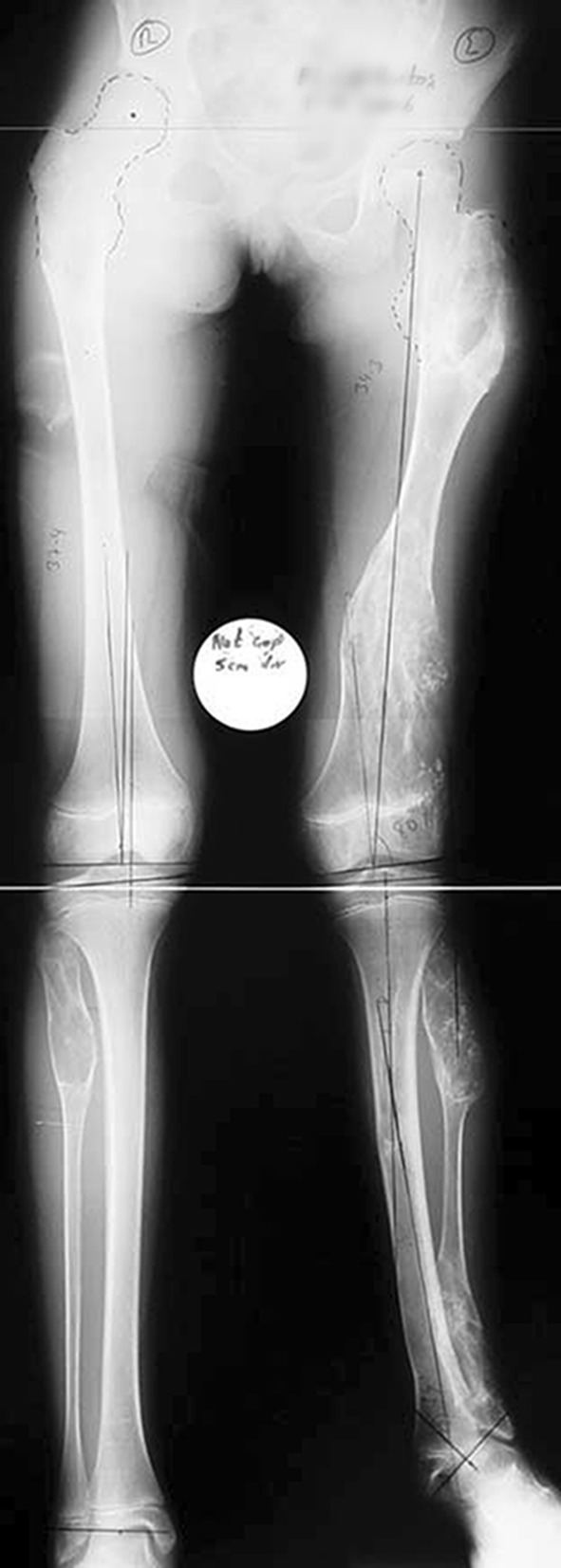
An eight-year-old girl with Ollier’s disease Lt. femur and tibia who developed valgus deformity and shortening following initial surgery of excision. Preoperative orthoroentgenogram denoting the LLD and the valgus deformity

**Fig. 2:**
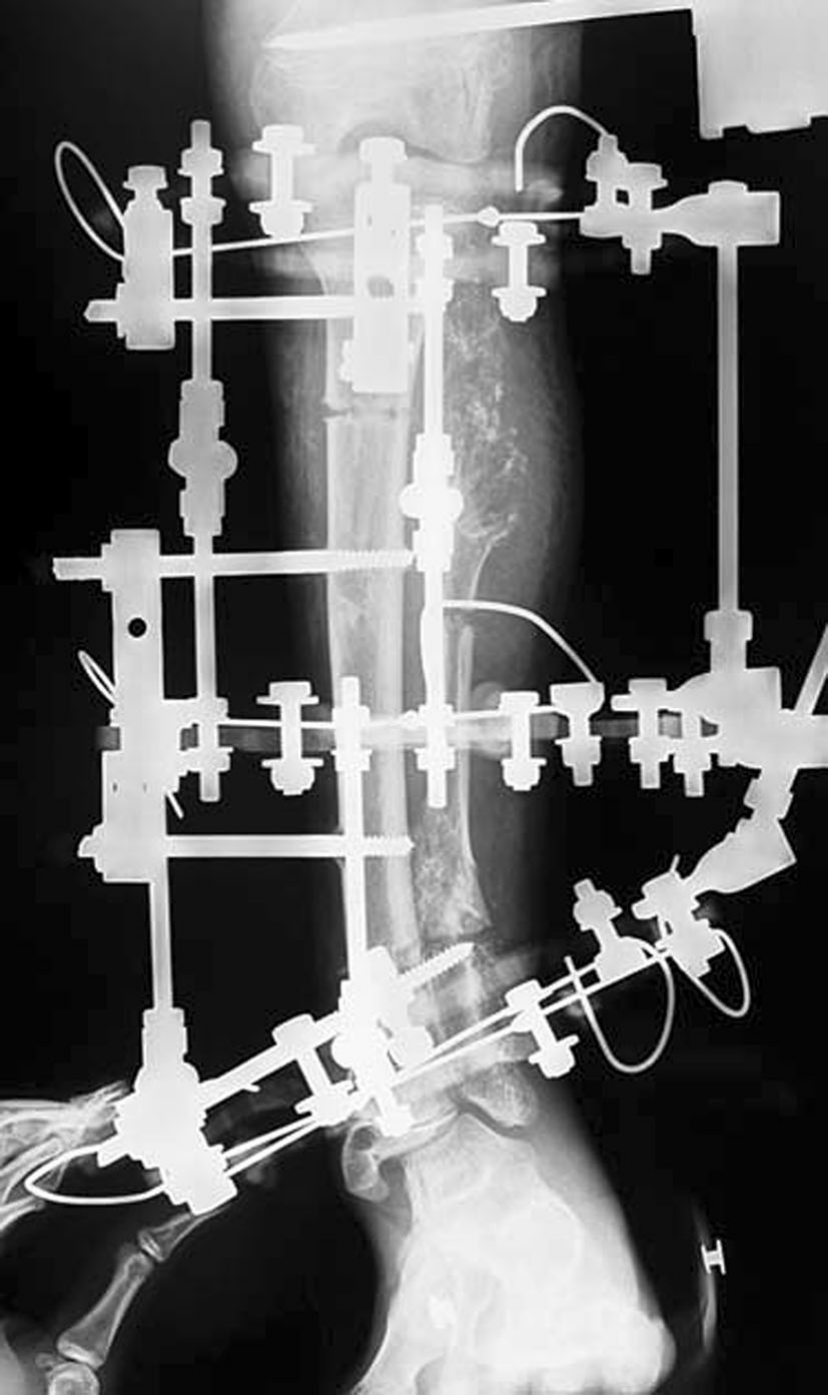
Tibial Ilizarov with proximal osteotomy for gradual lengthening and distal osteotomy for gradual correction of valgus

**Fig. 3:**
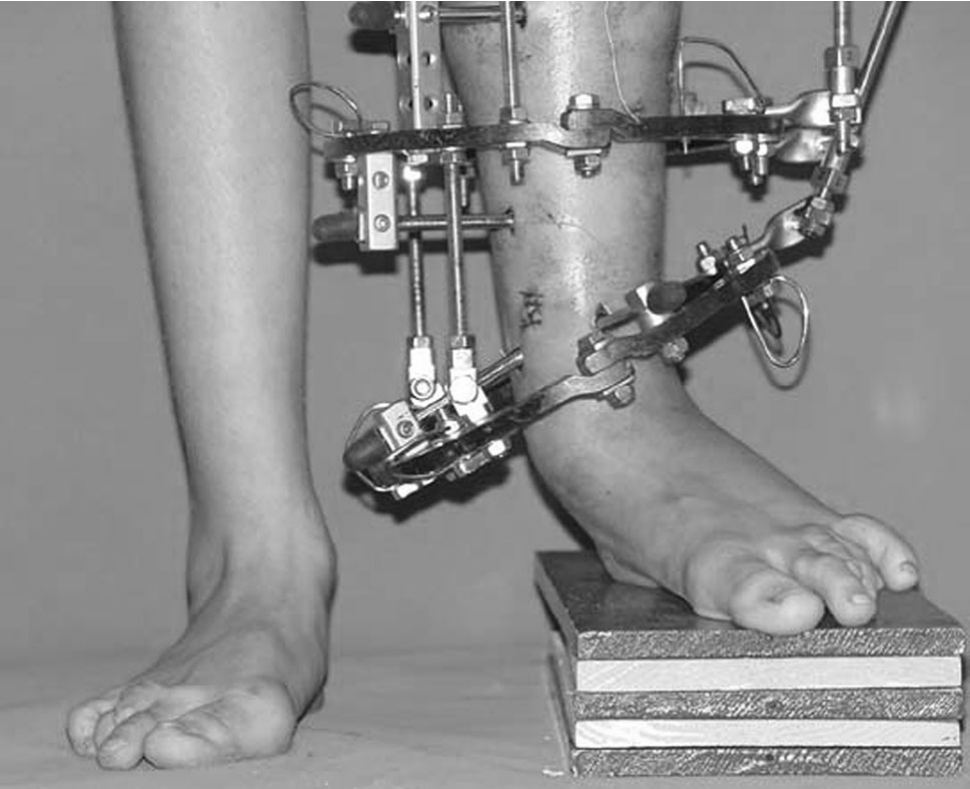
Clinical photo of the Ilizarov frame

**Fig. 4:**
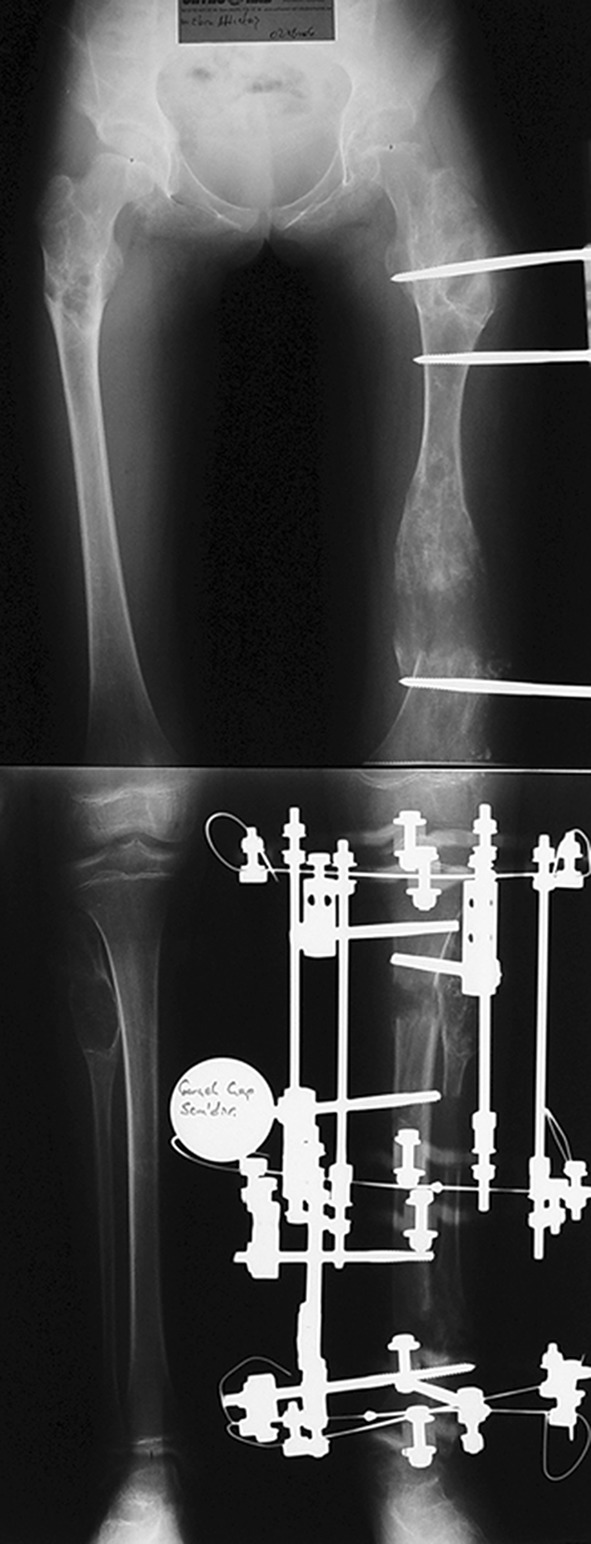
Femoral unilateral fixator for acute deformity correction and gradual lengthening, note the consolidation of the regenerates with corrected deformity

**Fig. 5:**
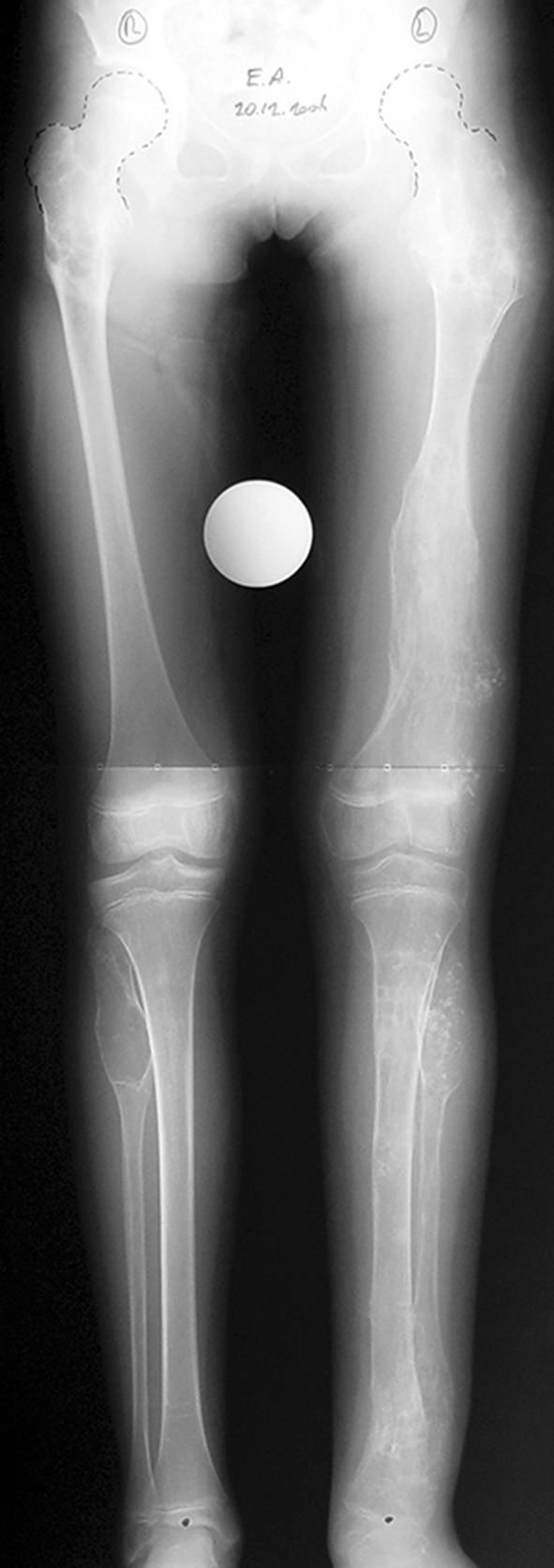
After removal of the fixators, restored length with deformity correction

**Fig. 6:**
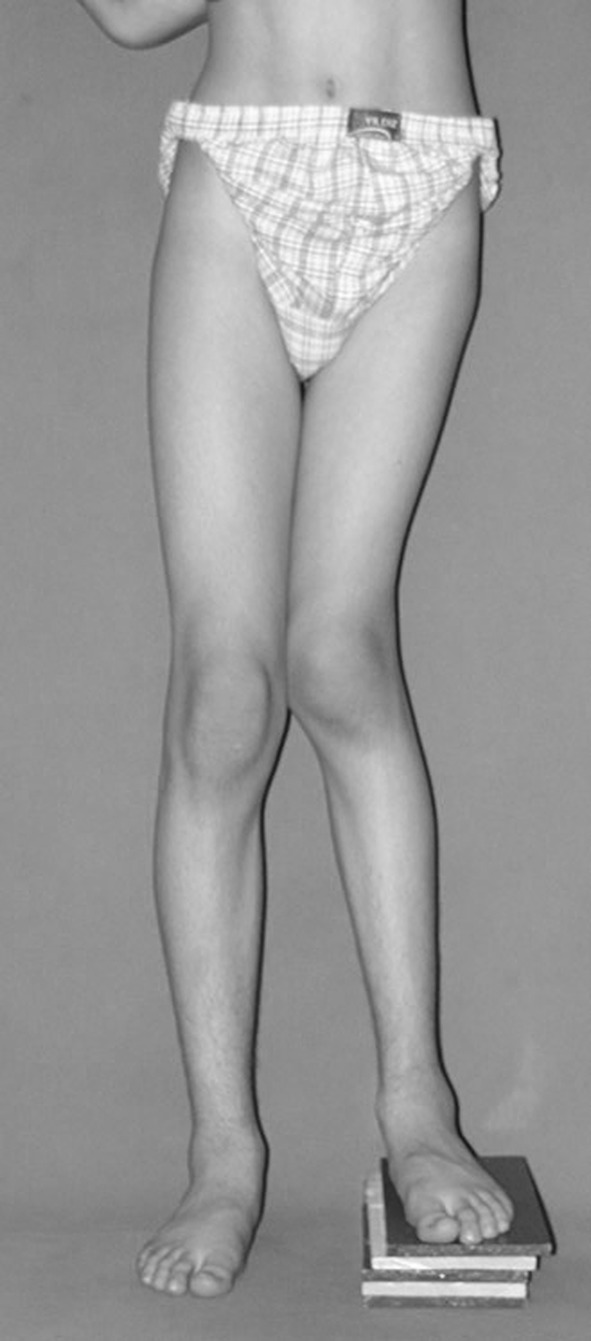
A thirteen-year-old boy with Fibrous dysplasia Lt. distal femur treated initially by excision. Clinically, block test denoting 5 cm shortening

**Fig. 7:**
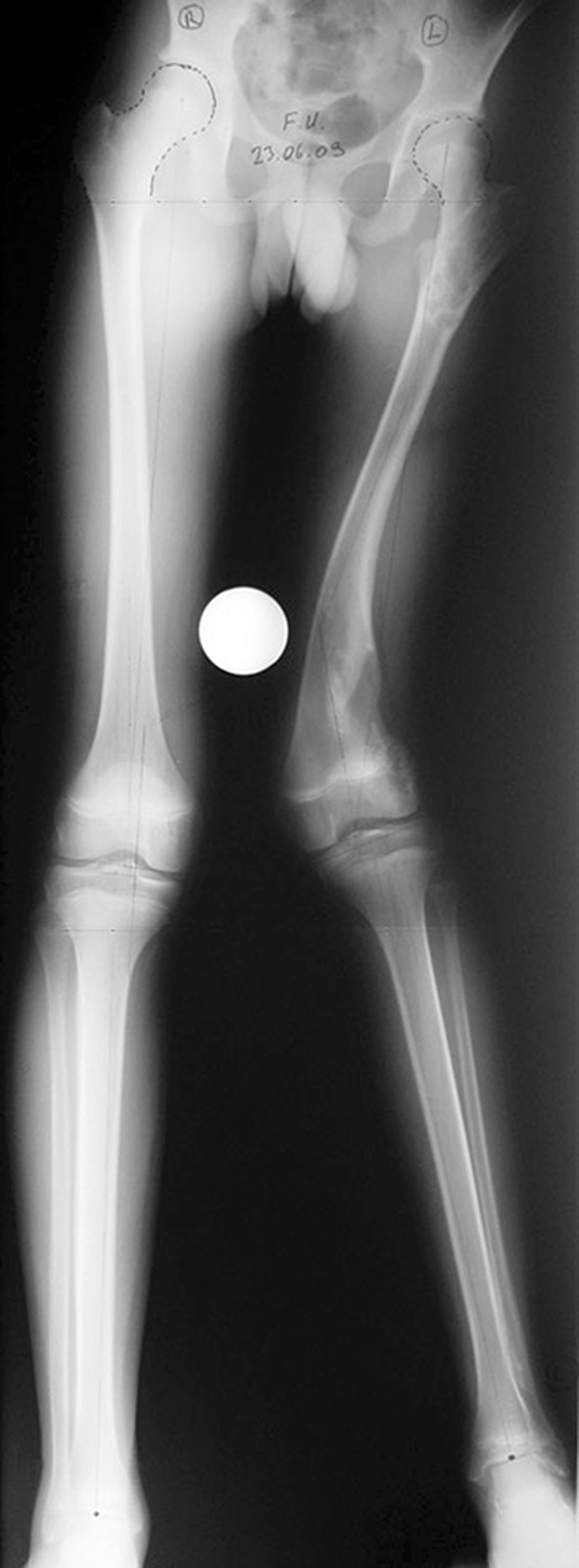
AP orthoroentgenogram, denoting valgus deformity with the CORA at the site of previous initial treatment

**Fig. 8:**
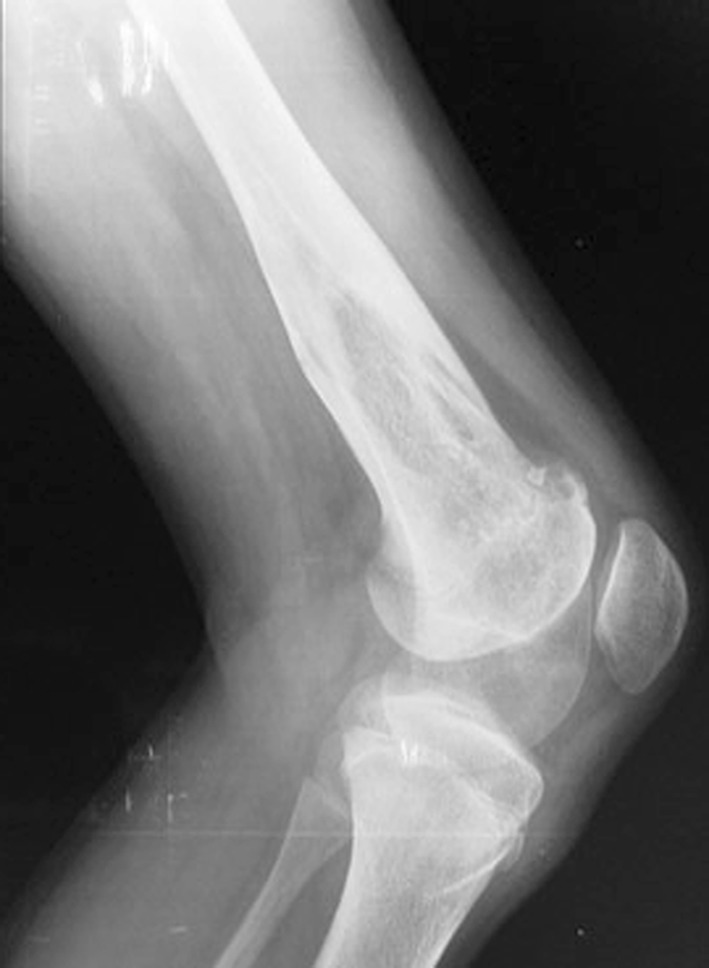
Lateral X-ray of the same patient

**Fig. 9:**
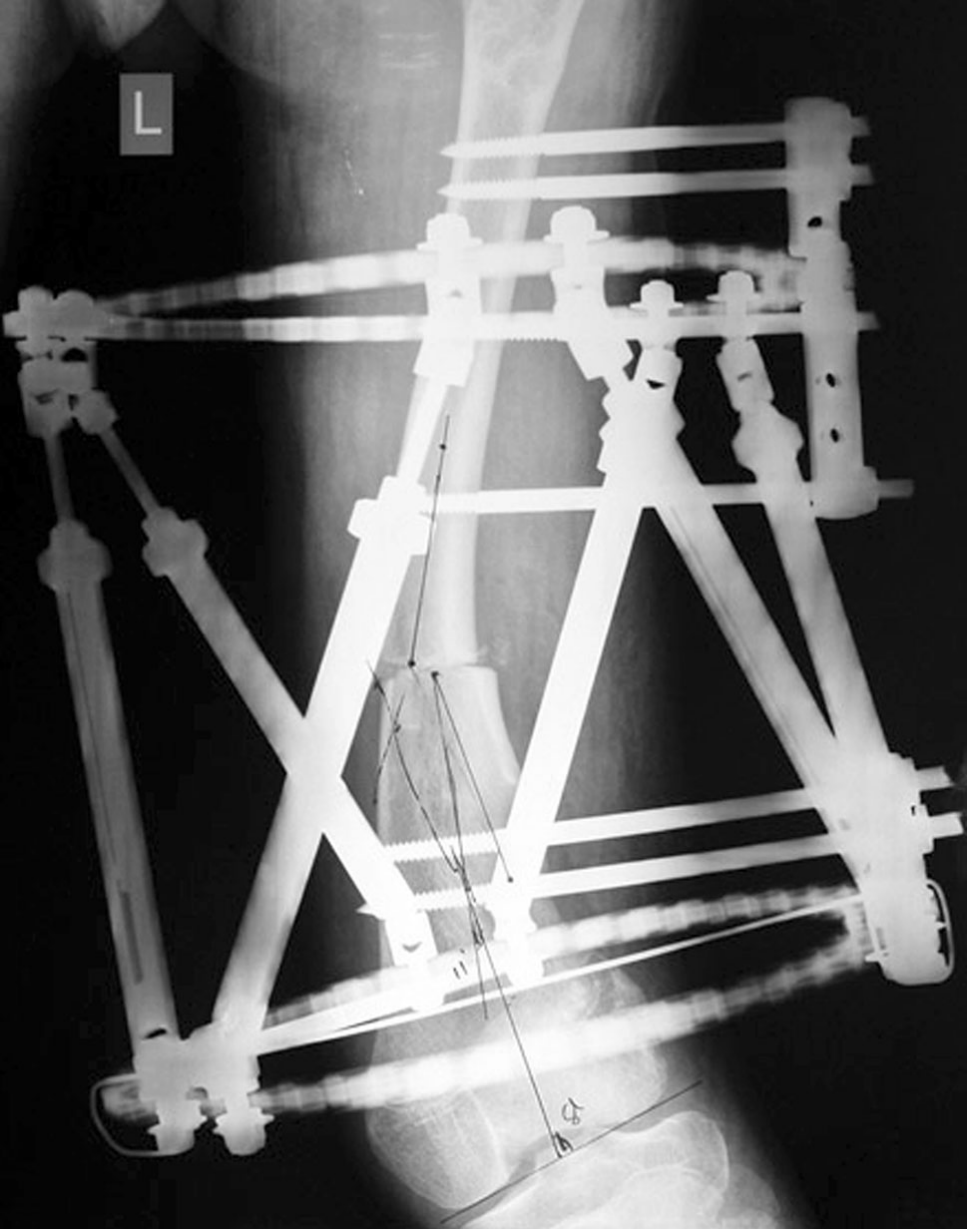
Immediately after the operation, with Smart correction multiaxial frame and distal femoral osteotomy

**Fig. 10:**
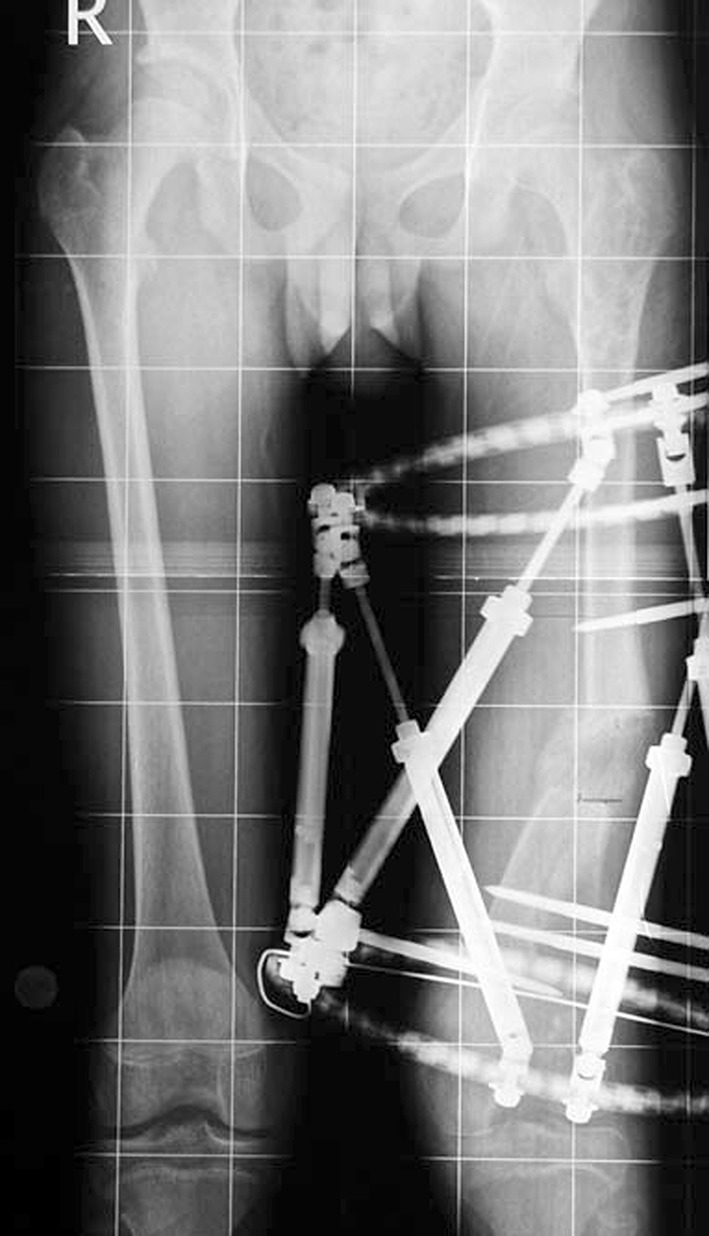
After correction and lengthening with good regenerate (note the amount of translation as the osteotomy site is not at the CORA)

**Fig. 11:**
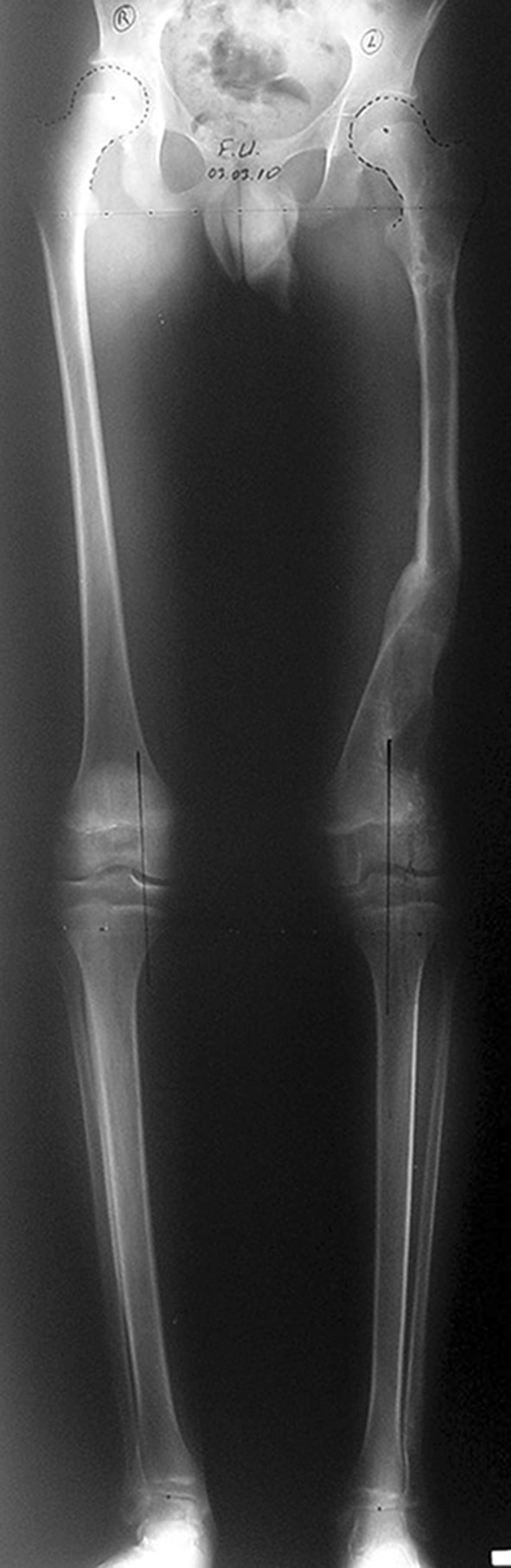
Orthoroentgenogram after removal of the frame with healed regenerate and fully corrected limb

**Fig. 12:**
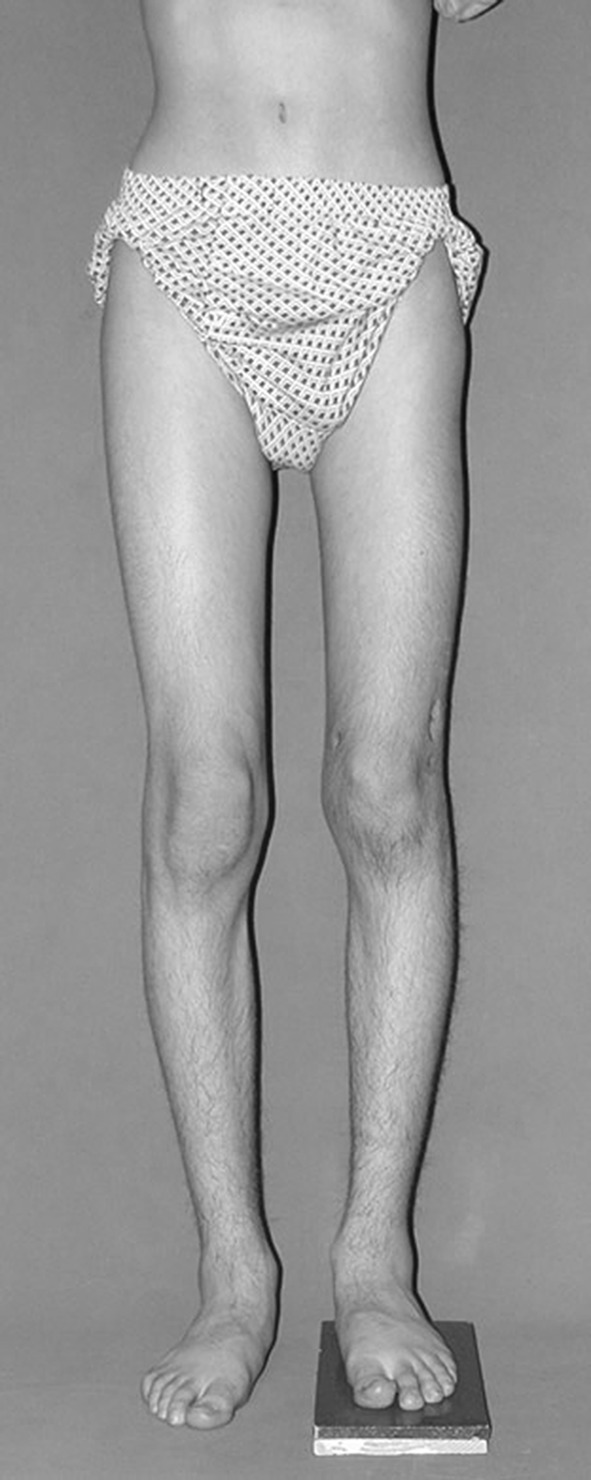
Clinically straight limb with the block test denoting residual shortening

**Fig. 13:**
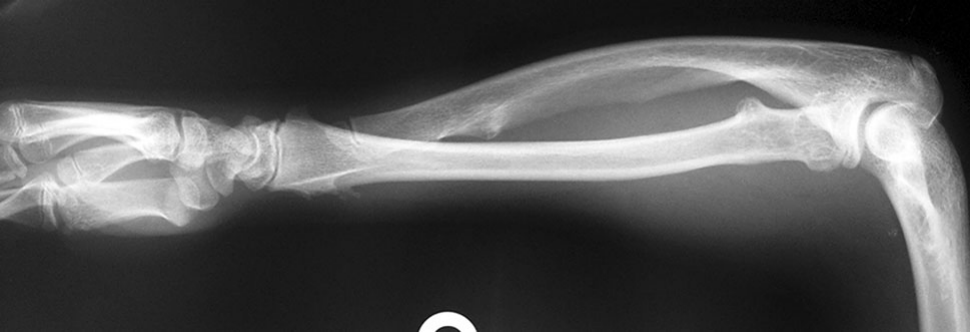
A fourteen-year-old boy with Congenital Multiple Exostosis Rt. Ulna treated initially by excision. X-ray showing type 1 deformity in which there is ulnar deviation of the hand and deformity of the radius

**Fig. 14:**
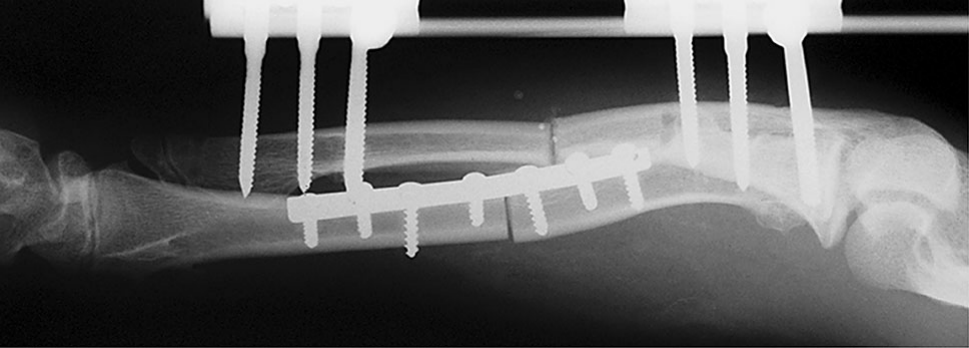
Acutely corrected radius by plate and screws. Also unilateral fixator in the ulna for gradual lengthening

**Fig. 15:**
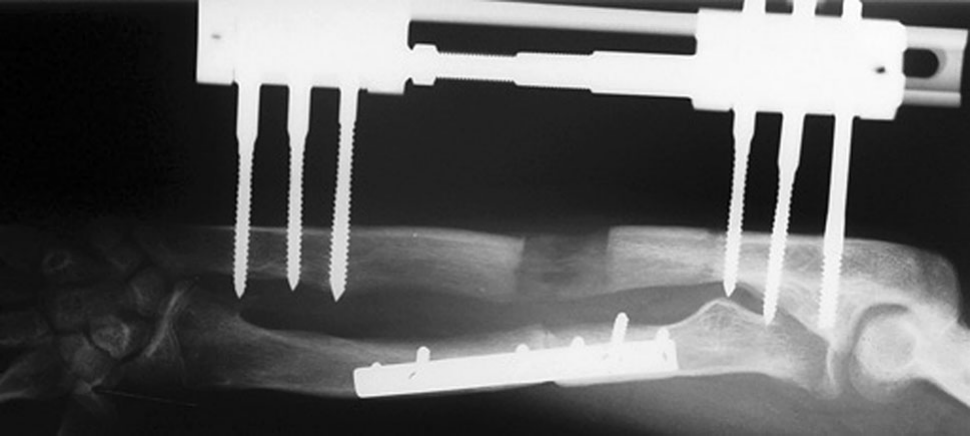
X-ray at the end of lengthening

**Fig. 16:**
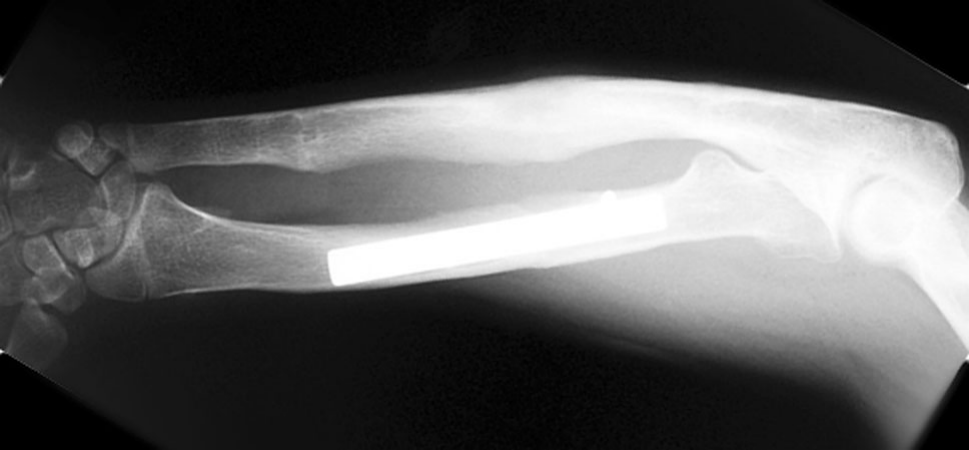
X-ray after removal of the external fixator, fully corrected deformity with excellent regenerate (note the 0.5 over-lengthening to avoid complications of recurrence and to improve the function

**Fig. 17:**
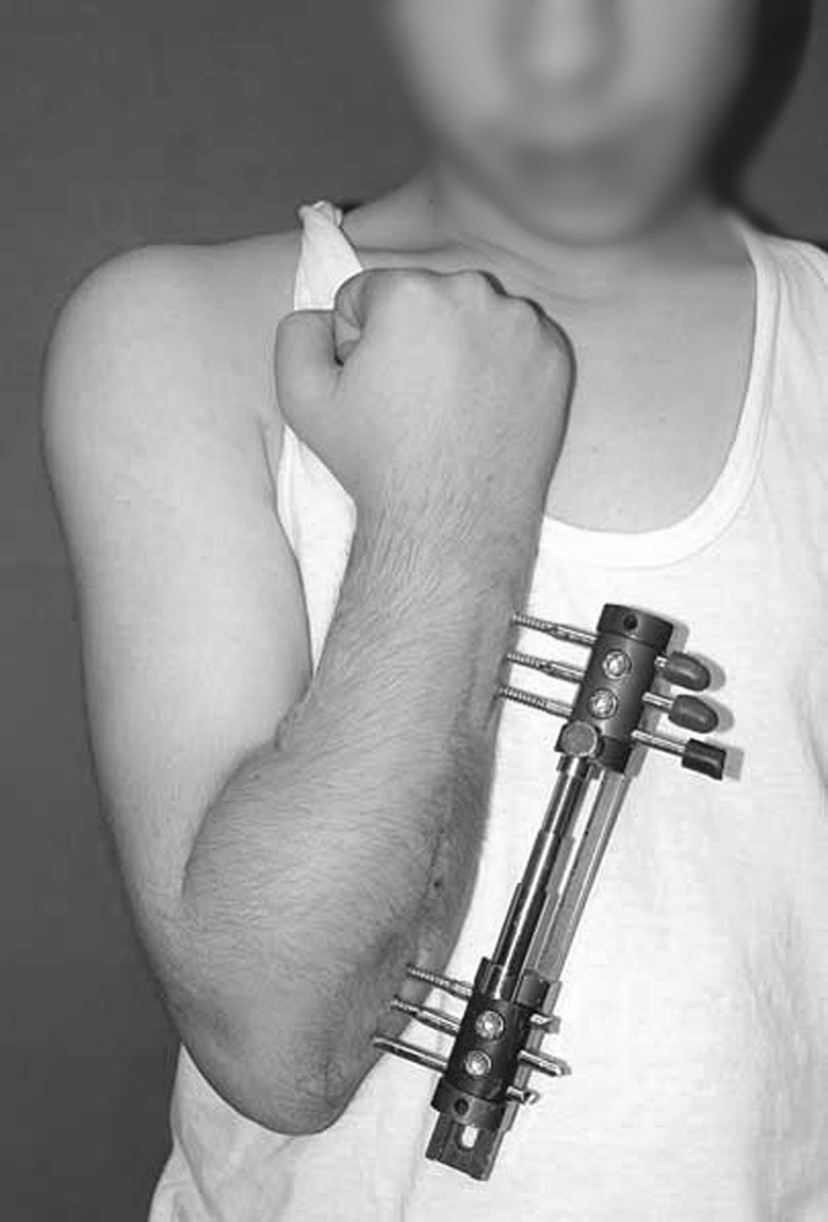
Photographic documentation during external fixation period

**Fig. 18:**
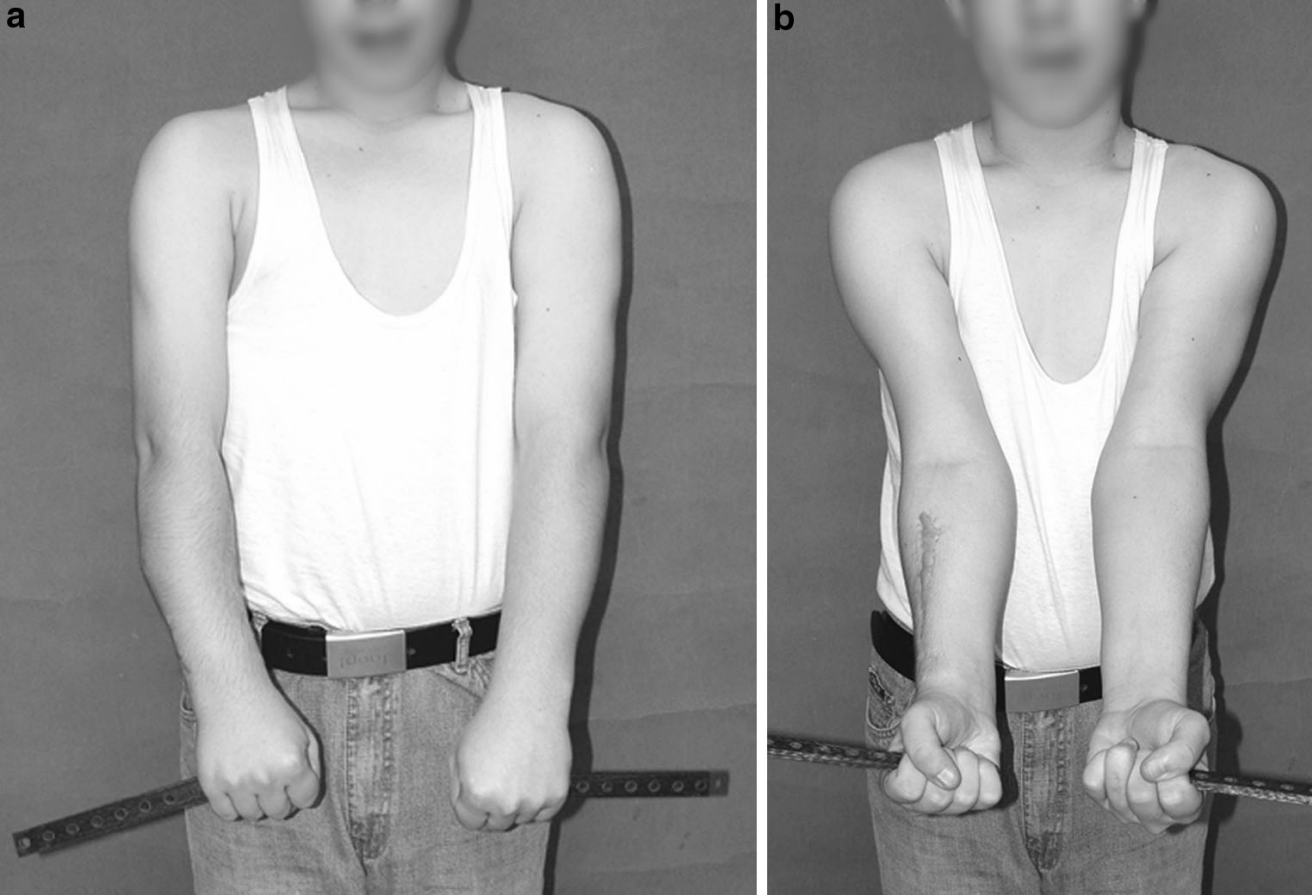
**a**, **b** Photographic documentation denoting fully corrected deformity and functional limb

**Table 1:** Patient demographic data

Patient groups (according to histological diagnosis)	Number of limbs	Mean age (range, years)	Location	Number of external fixators	External fixation time in days (mean, range)	Lengthening	Deformity correction plus lengthening	Bone transport
Ollier’s Disease	6	9.5 (7–14)	Femur 3 Tibia 2 Humerus 1	Ilizarov 1 Unilateral Fixator 1 MAC 1 EBI 1 TSF 1 Smart 1	168 (129–210)		5	1
Fibrous dysplasia	8	26.8 (11–58)	Femur 4 Tibia 4	TSF 7 Smart 1	123.8 (51–152)		8	
Congenital multiple exostosis	6	12 (10–14)	Femur 2 Ulna 4	Unilateral Fixator 4 Smart 2	201 (105–300)		6	
Giant cell tumour	2	22.5 (15–30)	Tibia 1 Radius 1	Ilizarov 1 Unilateral Fixator 1	148.5 (117–180)	1	1	
Desmoid fibroma	1	21	Tibia 1 + Gluteal region	Ilizarov & IM nail 1	90	1		
Chondromyxoid fibroma	1	22	Acetabulum 1	Ilizarov 1	270	1		
Chondroma	1	13	Femur 1	Ilizarov 1	210		1	
Unicameral bone cyst	1	14	Fibula 1	EBI 1	27		1	

*MAC* Multi-Axial Correction monolateral external fixation system (Biomet, Parsippany, NJ, USA), *EBI* External fixators (Dynafix; EBI, Parsippany, NJ, USA), *TSF* Taylor Spatial Frame (Smith & Nephew, Memphis, TN, USA) Smart Correction Multiaxial Frame: computer assisted circular fixator system (Response Ortho, USA)

A variety of fixation and reconstructive devices were used to accomplish the objectives of surgery. Limb lengthening was performed in three segments (one femur was treated using the Ilizarov fixator, one tibia was treated using the Ilizarov fixator with IM nailing and one tibia using the Ilizarov fixator combined with ipsilateral femoral IMN, both to compensate for limb discrepancy resulting after primary tumour excision). Combined limb lengthening with deformity correction was performed in 22 segments (22/26). We used the Taylor Spatial Frame (TSF) (Smith & Nephew, Memphis, TN, USA) to treat three femurs and five tibias, and Smart correction (computer assisted circular fixator system, Response Ortho, USA) to treat four femurs. A unilateral fixator was applied to treat one femur, one radius and four ulnas; Steinman pins were used as intramedullary devices for two ulnar cases. EBI external fixators (Dynafix; EBI, Parsippany, NJ, USA) were used to treat one fibula and one humerus, the Ilizarov fixator to treat one femur and a Multi-Axial Correction (MAC) monolateral external fixator (Biomet, Parsippany, NJ, USA) for one femur. Bone transport was performed in one limb (the Ilizarov device was used to treat tibia by bifocal compression distraction) (Table 1).

Prophylactic antibiotics were given to all patients for 2 days post-operatively. Distraction at the osteotomy site was often started 7 days post-operatively, at a rate of 0.25 mm every 6 h, with radiographs every 2 weeks. A rehabilitation programme of muscle and joint exercises was initiated immediately after surgery.

The mean follow-up time was 69.5 months (range 35–108 months). A functional assessment was done using criteria described by Paley et al. [15]. They are substantial limp, equinus rigidity of the ankle, soft tissue dystrophy (skin hypersensitivity, insensitivity of the sole, or decubitus ulcer), pain and inactivity (unemployment because of the leg injury or an inability to return to daily activities because of the leg injury). The results were considered excellent when the patient was active and had none of the other four criteria, good when the patient was active and had one or two of the other four criteria, fair when the patient was active and had three or four of the other criteria or had had an amputation, and poor when the patient is inactive or had five criteria.

## Results

The mean external fixation time was 159.5 days (range 27–300 days): 168 days in the OD group (129–210); 123.8 days in the FD group (51–152); 201 days in the CME group (105–300); 148.5 days in the GCT group (117–180); 90 days in the DF patient; 270 days in the CMF patient; 210 days in the chondroma patient; 27 days in the UBC patient. The mean length of distraction was 4.9 cm (range 0.2–14 cm) (Tables 2, 3, 4, 5). This gave a mean external fixation index of 67.4 days/cm (12–610) in 26 limbs that underwent distraction osteogenesis. This was 31.8 days/cm in the OD group (12–55), 140.4 days/cm in the FD group (14–610), 62.2 days/cm in the CME group (43–108), 28.5 days/cm in the GCT group (24–33), 26 days in the DF patient, 60 days in the CMF patient, 25 days in the chondroma patient and 39 days in the UBC patient.

**Table 2:** Patients with Ollier’s disease

Patient and age (years)	Site	(a) Initial treatment (b) Complication	Final treatment	EFT (days)	(a) Shortening (b) Lengthening (cm)	EFI (days/cm)	End result
F8	L Tibia and femur	(a) Excision (b) Shortness and deformity	Tibia bifocal compression distraction (Ilizarov), Femur deformity correction and lengthening (Unilateral fixator)	210	(a) 9.5 (b) 9	23	Def. corrected Res. shortening
F 14	R Femur and tibia	(a) Osteotomy distal femur and tibia (b) Deformity	MAC Frame for Femur TSF for Tibia	129	(a) 3.9 (b) 3.5	37	Def. corrected Res. shortening Knee joint contracture (resolved with PT)
F9	R Humerus	(a) Corrective osteotomy (b) Deformity	EBI frame	168	(a) 14 (b) 14	12	Def. corrected
F7	R Femur	(a) Biopsy (b) Pathologic fracture, shortening and deformity	Deformity correction and lengthening (Smart Correction multiaxial fixator)	165	(a) 4 (b) 3	55	Def. corrected Res. shortening

*EFT* External Fixation Time (in days), *EFI* External Fixation Index (in days/cm), *PT* Physiotherapy

**Table 3:** Patients with Fibrous dysplasia

Patient and age (years)	Site	(a) Initial treatment (b) Complication	Final treatment	EFT (days)	(a) Shortening (b) Lengthening (cm)	EFI (days/cm)	End result
M11	L Distal tibia	(a) Excision for recurrence, bone grafting, 8 mm fibular resection (b) Nonunion, recurrence and pin track inf	TSF	152	(a) 8.5 (b) 6.2	25	Def. corrected Res. shortening
F23	R&L Femur and tibia	(a) Bilateral femur and tibia osteotomy (b) Deformity	TSF	122	(a) 1.2 (b) 0.2	610	Def. corrected Res. shortening
F58	R Tibia	(a) Valgus osteotomy (b) Deformity and shortening	TSF	144	(a) 14.7 (b) 8.6	17	Def. corrected Res. shortening
M29	L Proximal femur	(a) Excision (b) Ankle equinus-treated with PT	TSF	51	(a) 3.7 (b) 3.7	14	Def. corrected
M13	L Femur	(a) Excision (b) Deformity and shortening	Smart correction multiaxial fixator	150	(a) 5 (b) 4.2	36	Def. corrected Res. shortening

*EFT* External Fixation Time (in days), *EFI* External Fixation Index (in days/cm)

**Table 4:** Patients with congenital multiple exostosis

Patient and age (years)	Site	(a) Initial treatment (b) Complication	Final treatment	EFT (days)	(a) Shortening (b) Lengthening (cm)	EFI (days/cm)	End result
M 14	L Ulna	(a) Excision (b) Ulnar club hand	Corrective osteotomy of radius, ulnar lengthening (Unilateral fixator)	270	(a) 2 (b) 2.5	108	Def. corrected
M 14	R Ulna	(a) Excision (b) Ulnar club hand	Corrective osteotomy of radius, ulnar lengthening (Unilateral fixator)	120	(a) 2 (b) 2.5	48	Def. corrected
F 10	R&L Ulna	(a) Excision (b) Bilateral ulnar club hand	Ulnar lengthening over Steinman pins (Unilateral fixator)	210	(a) 3 (b) 3.5	60	Def. corrected
M 10	R Femur	(a) Excision (b) Pathologic fracture, genu varum, 10 cm shortening	Deformity correction and lengthening (Smart correction multiaxial fixator)	300	(a) 8 (b) 7	43	Def. corrected Res. shortening
M 12	L Femur	(a) Excision (b) Deformity and knee contracture	Deformity correction and lengthening (Smart correction multiaxial fixator)	105	(a) 3 (b) 2	52	Def. corrected Res. shortening

*EFT* External Fixation Time (in days), *EFI* External Fixation Index (in days/cm)

**Table 5:** All other patients

Diagnosis	Patient and age (years)	Site	(a) Initial treatment (b) Complication	Final treatment	EFT (days)	(a) Shortening (b) Lengthening (cm)	EFI (days/cm)	End result
GCT	M 30	L Tibia	(a) Excision and tumour prosthesis (b) Septic prosthesis failure	Implant removal Femur LON, Tibia Ilizarov	180	(a) 7.5 (b) 7.5	24	Knee arthrodesis
	F15	L Radius	(a) Wide resection and non vascularized fibula graft (b) Recurrence, osteomyelitis	Lengthening and deformity correction (Unilateral External Fixator)	117	(a) 3.5 (b) 3.5	33	Def. corrected
DF	M 21	Tibia and gluteal region	(a) Wide resection (b) Shortening and sciatic nerve palsy	Pantalar arthrodesis (Ilizarov and IM nail)	90	(a) 5.5 (b) 3.5	26	Res. shortening
CMF	M 22	L Acet.	(a) Wide resection (b) Shortening	Femur lengthening (Ilizarov)	270	(a) 6 (b) 4.5	60	Res. shortening
Chondroma	F 13	R Distal Femur	(a) Wide resection (b) Shortening and deformity	Lengthening and deformity correction (Ilizarov)	210	(a) 9 (b) 8.5	25	Def. corrected Res. shortening
UBC	F 14	R Distal Fibula	(a) Curettage and bone grafting (b) Deformity	EBI frame	27	(a) 0.7 (b) 0.7	39	Def. corrected

*EFT* External Fixation Time (in days), *EFI* External Fixation Index (in days/cm), *LON* Lengthening over nail

All 20 patients returned to normal daily activities without pain at final follow-up. Only one patient with the proximal tibial GCT had a knee arthrodesis due to sepsis and prosthesis failure following initial surgery.

All patients were evaluated as excellent (using Paley’s functional criteria) except one with DF who developed a foot drop from sciatic nerve injury after initial surgery of tumour excision; this was treated by pantalar arthrodesis. Complications encountered included pin track infections in 6 patients which was treated by oral antibiotics, residual shortening in 8 patients and diminished joint motion due to knee arthrodesis in one patient.

## Discussion

Benign bone tumours are diagnosed in the juvenile age group usually with the deformity and shortening encountered progressive. Correction of the deformity but ignoring the limb shortening does not provide for a fully functional extremity at maturity [16].

A number of surgical treatments are proposed for correction of deformity, limb-length equalization and reconstruction in patients with bone tumours [16]. In this series of patients with benign bone tumours, we were able to treat most problems using external fixators. The aim was to achieve normal physiological alignment at maturity, and this may prompt a need for overcorrection and or overlengthening with distraction osteogenesis and the Ilizarov method. Currently, whilst different devices are used for this objective, the underlying principles are unchanged [16].

Multiple enchondromatosis (Ollier’s disease) is a common intraosseous benign cartilaginous tumour that develops in close proximity to the growth plate. It can cause deformity and limb-length discrepancy and carries a risk of malignant change to chondrosarcoma [17]. Conventional treatment is curettage and bone grafting which may result in severe deformities requiring repeated osteotomies. It is often difficult to obtain adequate stabilization and normal bone growth by autogenous bone grafting. Jesus-Garcia et al. [18] reported the use of the Ilizarov method in ten patients with Ollier’s disease. They reported excellent results and claimed the technique led to conversion of the abnormal cartilage to histologically mature bone in all their patients [18]. In this series all cases had accurate deformity correction with stable and mature bony regenerate. Three of the four cases had residual shortening (0.5 cm) that was not significant. One developed a knee contracture that resolved with physiotherapy. In spite of lengthening, which for some cases was over 9 cm and up to 14 cm, all four patients had excellent bony healing illustrated by the low EFI values (12, 23, 37 and 55 days/cm).

In fibrous dysplasia, curettage of the lesion and bone grafting may be effective for monostotic lesions but not for polyostotic fibrous dysplasia [19–21]. If the fibrous material is curetted and replaced by autogenous bone chips, these chips are often resorbed [22]. Curettage and bone grafting is not suitable in patients with deformity and pathological fracture. Corrective osteotomy with plate and screw fixation is relatively simple, but it can be difficult to achieve sufficient stability in fixation with screws in weakened bone; additionally, a fracture may occur because of stress shielding at the distal end of the plate [23]. Radical excisional surgery of the dysplastic bone will result in deformity frequently and lead to functional losses that can be of greater damage to the patient than the disease itself. [24] In this series, surgical lengthening and alignment of the mechanical axis was effective in preventing recurrent deformity and fracture. Of the five patients, four had residual shortening (Fig. 12). Three cases developed pin track infection during treatment which resolved completely using oral antibiotic therapy. The EFI in the FD group is high (140.4 days/cm). The external fixation time and index can be decreased using a combined technique such as an intramedullary nail and external fixator, but the consistency of the fibrous lesions in this condition may lead to technical difficulties in reaming and inserting an intramedullary nail into a long bone.

Congenital multiple exostosis (CMO) is characterized by growths of multiple osteochondromas (benign cartilage-capped bone tumours that grow outward from the metaphyses of long bones). Osteochondromas can be associated with an inhibition of skeletal growth, development of bony deformities, restricted joint motion, shortened stature, premature osteoarthrosis, and compression of peripheral nerves. Most individuals with CMO have at least one operative procedure and many have multiple procedures [25]. Femoral or tibial involvement often requires surgical deformity correction and lengthening. Early surgical treatment of tibio-talar tilt may prevent or decrease the incidence of late deterioration of ankle function, but long-term follow-up studies are needed to confirm [26]. Surgery for forearm deformity may involve excision of the osteochondromas, corrective osteotomies, and or ulnar lengthening procedures that may improve pronation, supination, and forearm alignment [27]. Radial hemiepiphyseal stapling, used alone or with ulnar lengthening, has been effective but causes unacceptable shortening of the forearm and the final result unpredictable [28]. In this series, the main problems in the three cases included ulnar deviation of the hand and deformity of the radius. After prior resection of the osteochondroma, ulnar lengthening was carried out with an external unilateral fixator concomitant with a corrective osteotomy of the radius with plate and screw fixation; there were satisfactory results and complete restoration of length of the ulna. We opted to perform over-lengthening by 0.5 cm in all the three cases to avoid recurrence of the ulna-radial length mismatch and to maintain improved function for longer (Fig. 16). One of these patients developed a recurrent radial deformity. The last two patients in this group had, in addition, femoral deformity and shortening which were treated successfully with using the Smart correction multiaxial fixator. One centimetre of residual shortening resulted in both cases.

The approach to treating giant cell tumours (GCT) has remained unchanged partly due to the lack of randomized clinical trials [29]. Surgery is the treatment of choice if the tumour is determined to be resectable. A number of strategies have been advocated including: curettage and grafting with autogenous bone graft; allograft or synthetic bone substitutes; either graft alone or combined with adjuvant therapy such as cryotherapy or the application of phenol after curettage [30–34]. Curettage is the commonly used technique [35], but it has reported recurrence rates of 27–55 % [36]. This high rate of recurrence is likely from an inadequate tumour resection rather than the use of adjuvant therapy [38]. Nonetheless, the high risk of recurrence led several surgeons to replace bone graft packing of the lesion with Poly Methyl Methacrylate (PMMA). The PMMA technique, compared with bone grafting, offers the advantages of lack of donor-site morbidity, an unlimited supply, immediate structural stability, low cost and ease of use. In addition, the barium contained in the methylmethacrylate results in a radiopaque substance that sharply contrasts with the surrounding bone. Local recurrences are more readily apparent than in cases in which bone graft is used [35]. However, there has disadvantages such as a thermal effect on articular cartilage, degenerative arthritis and that PMMA is not a biological substrate [37]. In this series there were two patients with GCT. One lesion located in the proximal tibia was managed initially by resection and prosthetic replacement. This became infected and was removed and followed by a course of antibiotics. The limb was then salvaged and the bone defect treated by knee arthrodesis, tibial Ilizarov and femoral lengthening over nail (LON). The second lesion was located in the distal radius. The patient developed a recurrence and osteomyelitis in the fibular graft used in the primary treatment. This was treated with further resection and distraction osteogenesis until both length and deformity were corrected.

Meary et al. in their nineteen cases of desmoid fibroma of the limbs noticed a large number of recurrences after surgical excision [39]. They concluded that treatment based on surgical excision should be as extensive as possible which leads usually to deformity and shortening. There was one patient with a desmoid fibroma affecting gluteal region and the tibia. After initial tumour excision, the patient developed shortening and a sciatic nerve palsy. This complication was addressed using the Ilizarov fixator and an IM nail for tibial lengthening and a pantalar arthrodesis to correct the foot drop. A residual shortening of 2 cm was the end result.

Chondromyxoid fibroma (CMF) is one of the rarest of bone tumours, accounting for less than 1 % of all bone tumours. The tumour is more common in males and located mostly in the metaphyseal areas of the lower extremity [40]. The most common method of treating CMF is with curettage followed by autograft or allograft. Occasionally, additional chemicals, such as phenol or liquid nitrogen, are placed inside the bone cavity to try to reduce the risk of recurrence. Lersundi et al., in their thirty cases of CMF, concluded that tumours treated with curettage alone did less well than those that were packed with allograft bone or polymethylmethacrylate and those treated by excision did not recur [40]. There was one patient with CMF in the acetabulum in this series. Initial treatment of resection led to shortening which was treated by femoral lengthening using the Ilizarov fixator.

The chondroma is a self-limiting lesion that, in most of cases, heals spontaneously with no treatment required for asymptomatic lesions. However, if a pathological fracture occurs it is treated with curettage and bone grafting [41]. The patient from this series was treated initially by wide resection for a distal femoral chondroma. The Ilizarov fixator was applied for deformity correction and lengthening; a residual 0.5 cm shortening was the outcome.

Despite an extensive literature on the unicameral bone cyst (UBC), there remains an uncertainty regarding optimal treatment. Bensahel et al. [42] have stated the solitary bone cyst has not revealed all its secrets. Surgical therapy of a UBC may be divided into open and percutaneous procedures. Success is quite varied and the very definition of success has also varied amongst authors [43]. The initial treatment of the patient with UBC of the distal fibula in this series was of curettage and bone grafting, after which shortening and deformity occurred. We applied EBI monolateral fixator for lengthening and deformity correction.

There is a concern regarding the risk for malignant degeneration in patients when an osteotomy is performed in bone with a coexisting benign tumour [5]. Similarly, there are concerns over the quality of new bone formation during distraction osteogenesis in what is ‘diseased’ bone [5]. Despite these concerns, we did not encounter these problems during a mean follow-up of 69.5 months.

## Conclusion

There are advantages of using distraction osteogenesis in the treatment of problems and sequelae after primary treatment for benign bone tumours. The risks for recurrence of shortening and deformity in young patients may be minimized with overcorrection or over-lengthening. There appears to be no increased risk of malignant degeneration from osteotomy through diseased bone or there being low-quality regenerate bone at the distraction site. We believe that external fixation is an effective technique for treating defects, problems and complications related to benign bone tumours or the effects arising from wide excision of the primary lesion. It offers a good alternative to other conventional methods of management. There are some disadvantages to this technique such as pin track infection, the bulk and encumbrance of the fixator and the prolonged treatment period. The choice of external fixator is dictated by the complexity of problem and the anatomical location but, in general, the circular fixators are more suitable than the unilateral fixators for the simultaneous treatment of deformity and limb-length discrepancy.
