# Erratum to: External fixation reconstruction of the residual problems of benign bone tumours

**DOI:** 10.1007/s11751-016-0248-4

**Published:** 2016-03-18

**Authors:** Levent Eralp, F. Erkal Bilen, S. Robert Rozbruch, Mehmet Kocaoglu, Ahmed I. Hammouda

**Affiliations:** Department of Orthopaedics and Traumatology, Istanbul Faculty of Medicine, Istanbul University, 34390 Topkapi, Istanbul, Turkey; Department of Orthopaedics and Traumatology, Memorial Health Group, 34385 Okmeydani, Istanbul, Turkey; Hospital for Special Surgery, Limb Lengthening and Complex Reconstruction Service (LLCRS), Weill Cornell Medical College, Cornell University, 535 East 70th Street, New York, NY 10021 USA; Orthopedic Department, Faculty of Medicine, Al-Azhar University Hospitals, Nasr City, Cairo, 11884 Egypt

## Erratum to: Strat Traum Limb Recon DOI 10.1007/s11751-016-0244-8

In the original article, family name of one of the co-authors (Ahmed I. Hammouda) has been published incorrectly. The correct family name should be Hammouda.

